# What surgeons tell patients and what patients want to know before major cancer surgery: a qualitative study

**DOI:** 10.1186/s12885-016-2292-3

**Published:** 2016-03-31

**Authors:** Angus G. K. McNair, F MacKichan, J. L. Donovan, S. T. Brookes, K. N. L. Avery, S. M. Griffin, T. Crosby, J. M. Blazeby

**Affiliations:** School of Social & Community Medicine, University of Bristol, 39 Whatley Road, Bristol, BS8 2PS UK; Severn School of Surgery, Deanery House, Unit D, Vantage Office Park, Old Gloucester Road, Hambrook, Bristol, BS16 1GW UK; Northern Oesophago-Gastric Unit, Royal Victoria Infirmary, Queen Victoria Road, Newcastle upon Tyne, NE1 4LP UK; Department of Oncology, Velindre Hospital, Whitchurch, Cardiff, CF14 2TL UK; University Hospitals Bristol NHS Foundation Trust, Bristol, BS2 8HW UK

**Keywords:** Communication, Oesophageal cancer, Surgical oncology, Informed consent

## Abstract

**Background:**

The information surgeons impart to patients and information patients want about surgery for cancer is important but rarely examined. This study explored information provided by surgeons and patient preferences for information in consultations in which surgery for oesophageal cancer surgery was discussed.

**Methods:**

Pre-operation consultations in which oesophagectomy was discussed were studied in three United Kingdom hospitals and patients were subsequently interviewed. Consultations and interviews were audio-recorded, transcribed in full and anonymized. Interviews elicited views about the information provided by surgeons and patients’ preferences for information. Thematic analysis of consultation-interview pairs was used to investigate similarities and differences in the information provided by surgeons and desired by patients.

**Results:**

Fifty two audio-recordings from 31 patients and 7 surgeons were obtained (25 consultations and 27 patient interviews). Six consultations were not recorded because of equipment failure and four patients declined an interview. Surgeons all provided consistent, extensive information on technical operative details and in-hospital surgical risks. Consultations rarely included discussion of the longer-term outcomes of surgery. Whilst patients accepted that information about surgery and risks was necessary, they really wanted details about long-term issues including recovery, impact on quality of life and survival.

**Conclusions:**

This study demonstrated a need for surgeons to provide information of importance to patients concerning the longer term outcomes of surgery. It is proposed that “core information sets” are developed, based on surgeons’ and patients’ views, to use as a minimum in consultations to initiate discussion and meet information needs prior to cancer surgery.

## Background

Interactions between surgeons and patients prior to undergoing operations are an important aspect of surgical oncology, although rarely the focus of research. The quality of these encounters matter because good communication is associated with better adjustment to illness, better quality of life (QOL), increased professional and patient satisfaction and fewer cases of litigation [1–5]. Furthermore, the information discussed in consultations forms the basis for informed consent for treatment, and patients look to clinicians to fulfil information needs [6, 7].

The level of detail that could be communicated before cancer treatments is vast, and it is unclear what information is critical to inform understanding in an individual [8–10]. Some patients want wide-ranging information [11, 12], whilst others prefer selected details [13, 14]. In practice, the disclosure of large amounts of information may result in ‘information overload’ and overly-long consultations. Patient-led communication, where discussions are guided by the individual, is helpful but patients may lack sufficient baseline knowledge to ask important questions. Within the context of information provision for surgery for cancer, it is necessary to communicate risks and benefits. Oesophagectomy is associated with mortality and morbidity and detrimental impact on QOL [15]. Adequate preoperative information is therefore essential, but little research has investigated information provision in this setting and it is currently difficult to know how much should be considered ‘enough’ [16]. This study explored verbal information provision by surgeons during pre-operative consultations, and patient preferences for information about oesophageal cancer surgery.

## Methods

This qualitative study comprised observations and interviews, and was conducted in three United Kingdom (UK) upper gastrointestinal (GI) cancer centres in 2010/11. Consultations between consultant surgeons and patients before surgery were audio-recorded to study information exchange, and semi-structured interviews were undertaken with patients within two weeks to explore views on the information provided and their preferences for information. Appropriate ethics committee approval was granted by the North Somerset and South Bristol Research Ethics Committee (project 07/H0106/185) and written informed consent gain from all participants.

### Participants

Eligible patients had oesophageal adenocarcinoma or squamous cell cancer and were selected for surgery alone, or neoadjuvant treatment and surgery by an upper gastrointestinal cancer multi-disciplinary team. Patients were eligible only when aware of results of diagnostic and staging investigations. Patients were excluded if a translator was required in the clinical consultation. All surgeons in the participating centres were eligible.

### Recruitment and data generation

Consecutive eligible patients were posted study information. Interested participants were met by researchers prior to a routine appointment in which treatment, including surgery, would be discussed by a surgeon. Consultations took place in usual hospital facilities.

Following the consultation, participants were invited to be interviewed at home, in the hospital or by telephone according to their choice. An interview topic guide was used to ensure that similar issues were covered in each interview, including expectations of the consultations, views on the information provided and information desired. This final topic included discussions about investigative tests, treatments, physical and psychological symptoms. The topic guide was applied in a flexible manner to allow patients to discuss issues of personal relevance. Interviews were conducted by FM, AGKM and JMB [17].

Data collection and analyses occurred concurrently and iteratively and the sample size was guided by assessment of the saturation of insights drawn from the data. Saturation was defined as the point at which no new relevant themes/subthemes were emerging from the iterative process of analysis. Patients’ clinical and demographic details were recorded, as were surgeons’ characteristics.

### Data analyses

Audio-recordings were anonymised and transcribed verbatim following standard notation guidelines [18]. Qualitative analysis software was used to assist with data management [19]. Analyses were undertaken by FM and AGKM and followed principles of thematic analysis [20]. Analyses of the consultation data focused identifying the topics covered and depth of details provided to patients by the surgeon, and patients’ responses to that information. Analyses of the interview data focused on exploring patients’ understanding of and views towards the information provided during the consultation and their preferences for information provision prior to cancer surgery.

Transcripts of consultations and interviews were read and re-read for data familiarisation, all transcripts of consultations and interviews were coded in an iterative process. Coding was partly theory driven, in that the focus of analysis was on information exchange and needs, but the researchers sought to ensure that themes emerged from the data. Researchers were aware literature describing cancer patients’ information needs [6], but they did not apply a priori categorisation to these data. Coding was conducted independently by two researchers (FM/AM) and a process of constant comparison used to compare transcripts. Codes were scrutinised and organised into broader thematic groups and sub-themes, where appropriate, which were then reviewed and discussed by other members of the research team (KNLA, JMB). Consultations and interviews were considered separately to explore patterns evident across multiple cases, and as paired consultations/interviews to explore the relationship between information provision during the consultation and the patient’s views and preferences. The plausibility of the data interpretation was discussed between the study team at regular interviews during the course of the analyses. Findings are presented in relation to the identified themes and frequencies are provided to illustrate the prevalence of themes.

## Results

Fifty two audio-recordings from 31 patients (mean age 67 years, 24 male; Table 1), comprising 25 consultations and 27 patient interviews were obtained. These characteristics broadly reflected those of patients routinely referred for oesophageal cancer surgery in the UK. Six consultations were not recorded because of equipment failure and four patients declined an interview. Consultations ranged from 9 to 41 min (mean 25), which reflected the 20 min time slot allocated to consultations by the institutions. No patients described the consultation as too brief. Interviews lasted between 10 and 78 min (mean 24) and most interviews (22) were in patients’ homes.

**Table 1:** Details of study participants (patients and surgeons)

Patient data	*N* = 31
Mean age, years (range)	67 (55–79)
Sex	
Male	24
Female	7
Tumour type	
Adenocarcinoma	18
Squamous cell cancer	13
Research centre	
1	24
2	2
3	5
Treatment stage	
Pre-chemotherapy & surgery	19
Pre surgery	12
Mean consultation length, minutes:seconds (range)	25.15 (09.74–41.37)
Surgeon data	*N* = 7
Sex	
Female	1
Male	6
Mean age, years (range)	47 (40–53)
Research centre	
1	5
2	1
3	1
Consultant experience	
> 5 years	5
< 5 years	2

Two main themes emerged: 1) surgeons emphasised surgical techniques and in-hospital risks, and 2) patients wanted information about post-operative recovery, long-term QOL and survival. An overview of the analysis is presented in Table 2.

**Table 2:** Summary of main themes and sub-themes

Theme	Sub-themes
Emphasis on surgical techniques and in-hospital risks by surgeons	Surgeons presented detailed technical information
	The gravity of the surgery was emphasized
	Short term risks were listed with little explanation
	Patients generally accepted the necessity of technical information
	Some patients did not want technical information
Post-operative recovery, long-term quality of life and survival were key patient information needs	Recovery and long-term quality of life information was desired by most, but not all, patients
	Long-term effects of surgery were minimised by surgeons
	Survival information was desired by patients
	Surgeons presented the uncertainty around survival
	Fear may inhibit patients’ desire for survival information

### Emphasis on surgical and in-hospital risks

#### Surgeons presented detailed technical information

All consultations were dominated by information from surgeons about operative technique and in-hospital morbidity risks. The information flow was unidirectional, with surgeons disclosing information *to* patients frequently in a uniform way with limited patient involvement. Descriptions were often detailed, and large amounts of information were communicated in a single discourse (Table 3). Information about operative technique followed a typical format involving an explanation of normal anatomy, identification of the tumour site, defining the extent of the resection and the method for reconstruction. Surgeons did not enquire if patients wanted this level of detail.

**Table 3:** Typical account of surgeons presenting detailed information on technique, and lists of risks, with minimal patient interaction. Patient utterances indicated in square brackets

*“All the tests suggest- you know, show this tumour in the lower oesophagus – there’s no obvious spread, as w- far as we can tell, to anywhere else in the body, so it’s confined to the lower oesophagus and perhaps the local lymph nodes. [Mm hm] Those get removed with surgery but involved lymph nodes is a worse, ultimate sign than if you didn’t have lymph nodes involved but only time will tell whether you’re lucky or you’re not.*	
*The surgical treatment involves removing the tumour and the oesophagus, so if this is the- if the tumour’s at the bottom of your oesophagus, we have to remove enough of the tumour- enough of the oesophagus for the stomach below to get- well, get it all out and then you’re left with a gap which, to be able to eat again, has to be put back together and what we do is we make a tube out of your stomach, like- freeing up the top bit of your stomach [Mm] and then that bit of the stomach is brought up into the chest to join onto the oesophagus, there, so it ends up looking a bit like this, so you’re diaphragm is here but your stomach is pulled up into your chest [Mm]. So, the operation involves an abdominal bit where we disconnect the top of your stomach from what’s attaching it in there, the bottom bit of the stomach stays where it is. We then turn you onto your side and go through your chest, collapse the lung so that we can see what we’re doing and then re-inflate the lung at the end of the operation and then pull the stomach up, make a tube out of it and join it to your oesophagus. So, that’s the technical side of the operation*	
*The bit that, er, causes the complication- well, it’s the complications afterwards that are- [Mm Mm] that are the what the potential problems and big operations have several complications – you can get chest infections, wound infections, you can get, er, bleeding, you can get heart problems, you can get, er, if that join we make leaks, that’s a serious complication [Mm], if the blood supply to the top of this bit of stomach’s not enough and then it dies, that’s a serious complication [Mm]- it clots in the legs. There are a whole range of things that are possible, the major it- you know, the majority of the people get through the surgery [Mm], erm and leave hospital so our mortality rate – the chance of dying in hospital from a serious complication is less than two percent, or around two percent [tut], so a ninety-eight percent chance of getting through major surgery [Mm]*	
**(Consultation with IS009)**	

#### The gravity of surgery was emphasised

The gravity of the surgery was emphasised, being described as ‘major’ or ‘big’ in 17 of the 25 consultations.

*“Now, the operation is a very big operation. It’s a very serious operation and there are risks involved, ok? It is one of the biggest operations a human being can actually undergo””* (consultant IS001).

Such descriptions allowed more detail about specific aspects of the procedure to be introduced, which reinforced the magnitude of the surgery may helped contextualise disclosure about in-hospital risks.

#### Short-term risks were listed with little explanation

Short-term risks were described in all consultations, and were listed in succession with little explanation (Table 3). The exception was in-hospital mortality, which often included summary statistics.

*“The overall mortality rate with a major operation like this, in our hands, is less than two percent, so it’s a ninety-eight percent chance of getting through it”* (consultation for IS010).

#### Patients generally accepted the necessity of technical information

Information about surgical technique and morbidity were identified as desired information topics by only three patients. Most patients acknowledged that surgeons needed to give them the data, and was often described in the context of possible litigation.

*“I think it’s, erm- ‘cause of litigation, isn’t it these days – they have to tell you everything” (*ISO001).

#### Some patients did not want technical information

There were seven patients that expressed a preference *against* being given technical information. This demonstrates a mismatch between surgeons’ and patients’ views. Explicitly not wanting to know about these things was potentially related to (a) a sense of inevitability about the procedure and a desire to ‘get on with it’:

*“I did have the fleeting thought going through my mind, ‘For goodness sake, why are you telling me all this. I’m confident, you’re confident. Let’s get on with it”* (IS015)

(b) that reflecting on their own vulnerability was unhelpful, and possibly contradicted a positive narrative that patients were trying to maintain

*“I don’t think I was as interested in that sort of detail. I know that there are risks, I don’t want to dwell on it. It’s always near the front of your mind at this particular time- and you’re trying to get away from that as much as possible* (IS017)

*“I must confess it came as rather a blow and what I what I didn’t like really were the statistics that he went into - I would have liked to have heard more about the sort of positive side of it”* (IS007)

or (c) a general squeamishness.

*“Surgeons see it every day. They’re quite happy to talk about it. A lot of people seen somebody run over in the road and their insides hanging out, they’d be on the side of the road throwing up. You know, and if they tell you they’re gonna do something similar to you, you don’t wanna know about it”* (IS002)

*“obviously one needs a- some idea of the process but not necessary of- not necessarily every gory detail”* (IS015)

### Recovery, quality of life and survival

Information about post-operative recovery and QOL was identified as important to all but four patients. This was related to a wide range of topics including work, social activities and physical symptoms.

*“I was trying to gauge what the time would be before I could begin to embark upon relatively normal activities”* (IS003)

*“Will I not be able to work any more?”* (IS004)

*“I wanted to know basically what you’re like. Can you, erm, do the things that I now do? Bearing in mind I’m seventy-six years old and I can’t run about like I used to…after six months, erm, how - what will it do? Can I- Will I be able to stretch? Will I be able to paint the ceiling- Will I be able to- to run about? What? I’ll be like- I’ll be able to drive a car, I guess but- you know, so those are the things.”* (IS013)

There were four patients who explicitly stated that they did not want information about QOL. Reasons for this included wanting the information later in their recovery or to maintain an idea of “hope”.

*“I don’t think that I would really want to know what would be the long-term problems if any. I want to stay on top – I want to keep on top of it… I don’t really want to think too far ahead, there is probably enough to think about, y’know, at the moment”* (IS008)

#### Long-term effects of surgery were minimized by surgeons

Long-term QOL were discussed in fewer than half (10) of consultations, with notable variation in the level of detail. Descriptions of recovery varied, from surgeons portraying it as an ongoing process, to describing a clear trajectory. Topics covered largely concerned the control of symptoms, such as reflux. Explicit in descriptions was that patients would return to a normal, or near-normal, state of functioning. This had the effect of minimising the long-term impact of surgery.

“*it can take six months or so before you are back to where you were, maybe longer—six to nine months to how you’re feeling now*” (consultation for IS019).

“*He said, ‘six months.’ But that’s to full fitness, you should be feeling a lot better a lot sooner*” (IS001)

Patients appeared satisfied with this information, though this may be based on the unrealistic belief that they would return to full health. Evidence suggests that half of patients never return to pre-operative levels of fitness [21], and this was not explained to patients. Minimising the long-term impact of surgery may therefore suppress question-asking. There were no examples of surgeons eliciting patients’ information needs regarding recovery.

#### Survival information was desired by patients

Survival information was often stressed as important by patients.

*“I’d like to know is- is your thoughts on, erm- on whether you’d like to know the- the chances of a successful cure and these kinds of things.* (ISO14)

It was provided in 17 consultations and quoted statistics were largely consistent between consultations and with published literature (50 % two year survival). Disclosure of survival information was often embedded within the technical description of the surgical procedure, and was brief.

#### Surgeons presented the uncertainty around survival

Although specific survival rates were conveyed in many consultations, surgeons made efforts to impress the uncertainty of the prognosis for the individual.

*“But, you know, as- as I s- tell people, you know, if- say there was a percentage cure rate, you’re not gonna be percentage cured, you’re either gonna be cured or not-[Yeah. Mm.] cured and that’s a problem – that’s when we just don’t know anything”*

These difficulties were manifested in consultations where survival statistics were often followed by caveats; “*we don’t have a crystal ball*” (IS028). This reflects tensions between providing population-based survival statistics and providing individualised information.

Difficulties with personalising survival information were acknowledged and largely accepted by patients during interviews, with uncertainty viewed as an inherent aspect of the cancer trajectory. This was even the case when such information was potentially distressing. In one interview the patient and his wife describe feeling ‘done down’ when hearing of the survival statistics, although the patient reflected; *“I thought, it’s better that [surgeon] said that than, ‘Oh look, we’ll cure you’”* (IS025).

#### Fear may inhibit patients’ desire for survival information

One patient initially described not wanting survival information but then clarified his opinion.

*“I’ve got to ask the question because clearly those are the answers you want to know, you know. Am I gonna die? Or, you know, how long am I likely to live? You know, these are sort of basic questions that you want answers to but you’re scared that someone’s gonna say well, actually not very long’, you know (laughs) and you can’t argue because they’re the professional”* (ISO7)

Fear was an inhibitory factor in this example but this highlights an important distinction between patients wanting survival information in general and wanting to know how long they will live as an individual.

## Discussion and conclusions

This study explored information provision by surgeons, and information desired by patients regarding surgery for oesophageal cancer. Analyses of consultation data found that surgeons consistently provided patients with detailed information about the surgical technique and associated in-hospital risks. Patient interviews showed that the information was accepted by patients as necessary but that patients wanted to know more about the long-term consequences of surgery. There is therefore a need to re-consider information routinely communicated before cancer surgery and to develop methods to ensure that appropriate operative details and short-term adverse events are explained, but also that information of key importance to patients is included.

Reasons for the observed differences between surgeons and patients’ views may be inferred from the different perspectives of the purpose of the consultation. Surgeons are aware of the medico-legal need to inform patients of risks, hence their detailed disclosure. They may be cautious to discuss survival, which is often poor in this patient group, and place less importance on the impact on QOL post-surgery. Whilst accepting information about these risks, patients are keen to hear about the potential benefits and the likely long-term consequences of surgery.

Some research has considered information preferences for patients undergoing oesophagectomy [7, 22–25], although there are no published studies that have used qualitative methodology to directly and in detail capture patients’ perspectives, observe real-life interaction with surgeons, or investigated needs for patients before undergoing surgery. A questionnaire study found that the majority of patients (79 %) wanted to know “as much as possible”, but further reasons about why this was the case were not explored [26]. Similarly, two studies examining patients’ preferences for prognostic information using discrete choice experiments demonstrated that the majority wanted to know survival rates, but broader information needs were not investigated [7, 23]. These studies were performed in patients who had undergone surgery, whose information preferences may have changed once the immediate peri-operative risks had been overcome. These studies highlighted patients’ need for information about postoperative QOL, and life style changes which may be particularly important in oesophageal cancer surgery [26]. Previous studies have demonstrated the difficulties of information provision in healthcare. Some authors suggested that detailed disclosure is desired by patients and recommended by experts [27–29], while others argue that patients express preferences for limited information, especially regarding ‘bad news’ [13, 14, 30]. Such tension was apparent in this study, with the majority (24) of patients extolling the value of knowing ‘as much as possible’, but a minority (7) holding an aversion to having some information disclosed to them. Rather than suggestive of a need for non-disclosure, these cases highlighted an obligation to provide information in a timely and sensitive manner over the course of the patient’s journey.

Deficiencies in surgical communication have been described in a recent systematic review [31]. Included were descriptive quantitative and qualitative studies that assessed communication behaviour of surgeons with patients and family members. At total of 31 papers were included describing 21 studies, the majority of which (25) were based in North America. The key findings of this review were largely in keeping with this research. Surgeons spent the majority of the time educating patients about surgery, but had deficiencies in discussing risks and uncertainties. Furthermore, little time was spent discussing “non-biomedical” issues. The main limitation of this review is with regards to the scope of surgery. Most of the studies focussed on low risk orthopaedic, gynaecological and general surgical procedures. Esophagectomy is one of the most morbid and mortal elective surgical procedures performed worldwide [32]. It represents patients only chance of long term survival, but the likelihood of achieving this is small. This study therefore represents an important and unique addition to this body of evidence.

This study is the first to provide detailed evidence of information provision and patients’ information preferences in a pre-oesophagectomy setting across several UK centres with paired observational and interview data, and succeeded despite major challenges. Research involving patients with oesophageal cancer is difficult because survival is short. Pre-operative recruitment is challenging as there is a narrow time window, and patients’ have much to consider without the burden of research participation. This work was based around one clinical episode that was chosen to represent the time when maximal information about surgery would be provided. It is likely that surgery was discussed at other times and with other professionals. It is possible that key information was imparted at those time points although patients did not highlight this issue during the individual interviews. Data in this study were presented comparing themes across the consultations and interviews because it best reflected the aims of the study. It would be interesting to present further data to explore paired consultations/interviews, however, this will be the subject of future research. Similarly, the use of other forms of data collection or analysis, such as video recording and conversation analysis, would provide further insights into the communication process.

Sampling in qualitative research may involve purposefully selecting relevant participants through ‘non-probability’ sampling to attain a broad range of possible views [33]. In this study, consecutive patients referred for surgery were invited to participate, using a ‘convenience sampling’ approach that may be considered less rigorous and produce findings that are potentially less generalizable [34] but may be deemed appropriate when the population is hard to access [35]. Patients in this study were sampled from three UK centres and their characteristics broadly reflected those of the population of patients with oesophageal cancer as a whole. Furthermore, the consistency and plausibility of the findings indicate that the findings may be transferable. Further work in a wider range of centres and different contexts, including those outside the UK, is encouraged to more fully understand the practices and patient preferences relating to information provision indifferent health care and medico-legal systems. Cultural differences, such as the primacy of individual autonomy or degree of professional beneficence would be worth exploring.

Information provision is central to clinical consultations. This study demonstrated a discrepancy between desired information and information provided to patients in surgical consultations. It is therefore necessary to develop methods to improve information provision in surgical consultations. One possible solution is to develop a “core disclosure set of information” [36]. This is a minimum information set agreed by both surgeons and patients that would act as a baseline to enable patients to consider their own preferences and stimulate shared decision- making. In that way it does not substitute individualised disclosure, but acts as an initial foundation to catalyse discussions that are meaningful to the patient. Benefits of such an approach are to avoid overloading patients, whilst still enabling participation in the disclosure process. Research is currently in progress to define core disclosure for oesophagectomy, using methods developed for defining outcome measures for trials, although how this will be integrated into clinical practice is still under development.

## Abbreviations

GI: Gatrointestinal
QOL: Quality of Life
UK: United Kingdom
